# Chitosan/Cyclodextrin Nanospheres for Potential Nose-to-Brain Targeting of Idebenone

**DOI:** 10.3390/ph15101206

**Published:** 2022-09-28

**Authors:** Federica De Gaetano, Nicola d’Avanzo, Antonia Mancuso, Anna De Gaetano, Giuseppe Paladini, Francesco Caridi, Valentina Venuti, Donatella Paolino, Cinzia Anna Ventura

**Affiliations:** 1 Department of Chemical, Biological, Pharmaceutical and Environmental Sciences, University of Messina, Viale Ferdinando Stagno D’Alcontres 31, I-98166 Messina, Italy; 2 Department of Pharmacy, University “G. D’annunzio” of Chieti-Pescara, Via dei Vestini, 31, I-66100 Chieti, Italy; 3 Department of Experimental and Clinical Medicine, University of Catanzaro “Magna Graecia”, Viale Europa s.n.c., I-88100 Catanzaro, Italy; 4 Department of Life Sciences, University of Modena, Via Dei Campi, 287, 41125 Modena, Italy; 5 Department of Mathematical and Computer Sciences, Physical Sciences and Earth Sciences, University of Messina, Viale Ferdinando Stagno D’Alcontres 31, I-98166 Messina, Italy

**Keywords:** idebenone, chitosan nanospheres, cyclodextrins, in vitro biological studies, FT-IR

## Abstract

Idebenone (IDE) is a powerful antioxidant that is potentially active towards cerebral diseases, but its low water solubility and fast first pass metabolism reduce its accumulation in the brain, making it ineffective. In this work, we developed cyclodextrin-based chitosan nanospheres (CS NPs) as potential carriers for nose-to-brain targeting of IDE. Sulfobutylether-β-cyclodextrin (SBE-β-CD) was used as a polyanion for chitosan (CS) and as a complexing agent for IDE, permitting its encapsulation into nanospheres (NPs) produced in an aqueous solution. Overloading NPs were obtained by adding the soluble IDE/hydroxypropyl-β-CD (IDE/HP-β-CD) inclusion complex into the CS or SBE-β-CD solutions. We obtained homogeneous CS NPs with a hydrodynamic radius of about 140 nm, positive zeta potential (about +28 mV), and good encapsulation efficiency and drug loading, particularly for overloaded NPs. A biphasic release of IDE, finished within 48 h, was observed from overloaded NPs, whilst non-overloaded CS NPs produced a prolonged release, without a burst effect. In vitro biological studies showed the ability of CS NPs to preserve the antioxidant activity of IDE on U373 culture cells. Furthermore, Fourier transform infrared spectroscopy (FT-IR) demonstrated the ability of CS NPs to interact with the excised bovine nasal mucosa, improving the permeation of the drug and potentially favoring its accumulation in the brain.

## 1. Introduction

**Figure 1:**
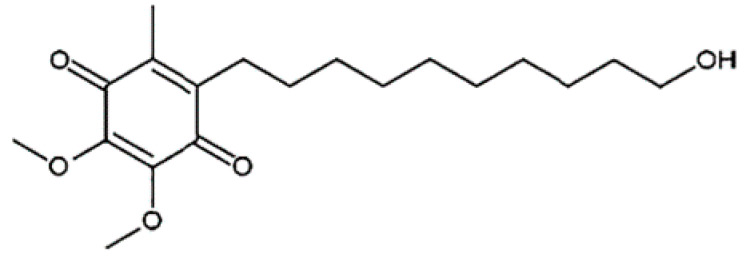
Chemical structure of IDE.

Idebenone (IDE), a synthetic short-chain analogue of coenzyme Q10 (Figure 1), is a potent antioxidant [1] that is able to prevent lipid peroxidation, thus protecting mitochondrial membrane integrity toward oxidative damage [2,3]. It can also accelerate the biosynthesis of ATP by activating the electron-transfer system of mitochondria, overcoming mitochondrial complex I and transferring electrons directly to mitochondrial complex III (by-passing complex I), hence reducing the consumption of non-respiratory oxygen [4,5]. Studies demonstrated that the drug is able to stimulate the synthesis of nerve growth factor and the recovery of choline acetyltransferase activity in basal forebrain injured rats [6]. Furthermore, Parkinson’s disease mice treated with IDE showed reduced dopaminergic neuron damage and improvement in behavioral disorders [7].

Nowadays, IDE is approved as an orphan drug, with the trademark Raxone^®^ (Santhera Pharmaceuticals, Pratteln, Switzerland), to treat visual impairment in subjects affected by Leber’s hereditary optic neuropathy (LHON), a rare disease based on genetic mitochondrial alteration that causes progressive vision loss [8,9]. Furthermore, IDE is on the Italian market (Mnesis^®^, Takeda Italia SpA, Rome, Italy), Turck (Bermaxin^®^, CELTİS İLAÇ SAN. VE TİC. A.Ş., Avrupa, Turkey and Everon^®^, NEUTEC İLAÇ SAN. TİC. A.Ş., Esenler/İstanbul, Turkey) and other markets, as mono- or pluri-component formulations, for the treatment of cognitive defects due to vascular or degenerative neurological diseases [10]. IDE, based on its antioxidant effects and influences on mitochondrial respiratory chain complexes, could be effective in the treatment of all conditions linked to mitochondrial dysfunctions. However, contradictory results have been obtained in different studies concerning the efficacy of IDE in the treatment of Friederich Ataxia (FA), Duchenne muscular dystrophy (DMD) and Alzheimer’s disease (AD), all characterized by a mitochondrial disfunction [11,12,13]. In 1986, IDE was authorized in Japan for AD treatment, but in 1998, the drug was withdrawn from the market due to clinical trials demonstrating lack of proven efficacy [14]. Subsequently, Santera Pharmaceuticals performed studies to evaluate the efficacy of IDE (Catena^®^, Pistoia, Italy) in reducing cardiac hypertrophy and in improving neurological functions in patients affected by FA [15,16,17]. However, after a first conditioned authorization, the drug was withdrawn from the market for lack of efficacy [18]. A similar fate was reserved to a phase III study (SIDEROS trial by Santera Pharmaceuticals) to evaluate the efficacy of IDE (Puldysa^®^, Santera Pharmaceuticals, Lörrach, Deutschland) in delaying the loss of respiratory function in Duchenne muscular dystrophy (DMD) patients assuming glucocorticoids. In 2020, the company announced the discontinuation of the study due to failures of their primary endpoints [19]. An insufficient concentration of IDE into the target tissues after oral administration of solid formulations (the only present on the market) could probably be responsible of the lack of IDE efficacy.

IDE exhibits a very low water solubility (0.8 mg/100 mL at 25 °C). After oral absorption, it is rapidly transformed by the first-pass metabolism into inactive metabolites [20] and a very low amount (about 1%) was detected in plasma [21]. Furthermore, the high affinity of IDE for plasma proteins could contribute to reduce the brain accumulation, thus negatively affecting its therapeutic potentialities. In light of the above considerations, alternative dosage forms are needed. Different approaches have been put in place to improve the water solubility of IDE, such as encapsulation into liposomes [22,23], complexation in cyclodextrins [24,25,26,27], realization of nanoroads [28]. In all cases, significant increase of water solubility and dissolution rate were obtained, that could permit the realization of liquid formulations, improving oral absorption of the drug. However, other strategies can more efficiently increase the distribution of IDE into the brain, making the treatment of specific cerebral defects more effective with respect to conventional forms. Intranasal administration of drug-loaded nanoparticles could be a valid approach to deliver the drug directly into the brain through the olfactory nerve pathway [29,30]. In this way, treatment of specific brain diseases, such as Alzheimer’s or Parkinson’s diseases, could be more efficacious than conventional administration routes. Studies demonstrated that chitosan nanospheres (CS NPs), administered through the nasal cavity, improve the brain accumulation of cargos due to their muco-adhesiveness and ability to open tight junctions in the olfactory and respiratory epithelia [31,32]. Furthermore, authors demonstrated their uptake by olfactory ensheathing cells [33,34]. CS is a hydrophilic, biocompatible polymer [35] that is widely studied as a polymer for sustained drugs or peptides released from microparticle or nanoparticle formulations [36,37,38,39,40,41]. Ionotropic gelation or polyelectrolyte complexation of positively-charged CS with different polyanions, such as tripolyphosphate, sodium sulphate, alginate, hyaluronate, and others, represents a simple and mild method to obtain NPs with low sizes, high loading, and good delivery ability for hydrophilic molecules [42]. The process is entirely performed in aqueous medium; thus, it is not suitable for the effective encapsulation of lipophilic molecules. Recently, authors demonstrated that anionic cyclodextrins (CDs), such as carboxymethyl-β-CD (CM-β-CD) and sulfobutylether-β-CD (SBE-β-CD), can electrostatically interact with CS to develop NPs for drug delivery [43,44,45,46]. Moreover, due to their hosting capacities, these CDs can include hydrophobic drugs [47,48,49,50], improving their physical–chemical properties (first of all, aqueous solubility). In this way, anionic CDs can act as gelling agents for CS, to prepare NPs, and in the meantime, they increase water solubility of lipophilic drugs, by complexation, permitting their encapsulation within CS NPs prepared in aqueous medium. Recently, we reported that sulfobutyl ether-β-cyclodextrin (SBE-β-CD), an anionic β-CD derivative approved by the FDA for intramuscular (IM) and intravenous (IV) administration, forms a stable inclusion complex with IDE, increasing its water solubility and dissolution rate [27]. Montenegro et al. [51] demonstrated that CS NPs obtained with SBE-β-CD loading IDE and glutathione possess high antioxidant activity and no toxicity in human keratinocytes, representing a good strategy for the design of formulations for topical treatments with antioxidants.

On this basis, in this work, we developed CS/SBE-β-CD NPs as potential carriers for nose-to-brain delivery of IDE. The NPs were prepared by ionotropic gelation of CS with the free SBE-β-CD or IDE/SBE-β-CD inclusion complex and were overloaded by using the soluble IDE/hydroxypropyl-β-CD (IDE/HP-β-CD) inclusion complex [24]. The systems were investigated regarding their physical–chemical, morphological, and technological (encapsulation parameters and release of the drug) properties. The tolerability of the carrier and its influence on antioxidant activity of IDE were assayed in vitro on human U373 glioblastoma cells. Ex vivo studies on excised bovine nasal mucosa were performed to evaluate the ability of NPs to improve the permeation of the drug. Finally, FT-IR analyses were carried out to obtain insight into the interactions of the carrier with the excised nasal mucosa, focusing on the reorganization of the hydrogen-bond (HB) network. 

## 2. Results and Discussion

Ionotropic gelation of CS with a polyanion is a process entirely carried out in aqueous solution, which produces NPs that are able to encapsulate (with high efficiency) just hydrophilic molecules [42]. To encapsulate a lipophilic molecule, its water solubility must be improved. In this work, we used SBE-β-CD, a negatively-charged CD, approved by the FDA for parenteral administration, to improve water solubility of IDE, by means of the formation of a soluble inclusion complex. In the meantime, SBE-β-CD acts as a polyanion for CS, permitting the formation of IDE loaded-CS NPs. The IDE/SBE-β-CD inclusion complex was prepared by a freeze-drying method, and this allowed us to obtain a solid complex able to dissolve immediately before the addition to the CS solution for NP preparation.

The addition of free SBE-β-CD to the CS solution produced NPs (empty NPs) with good properties in terms of hydrodynamic radius (R_H_), homogeneity (as accounted by polydispersity index (P.I.)), surface charge (as expressed by zeta potential, ζ), and yield percentage (Table 1). These properties were maintained by NPs produced by the gelation of CS with the IDE/SBE-β-CD inclusion complex; however, the yield percentage was sensibly reduced, probably because of the inclusion complex geometry. Our FTIR-ATR studies [27] demonstrated that IDE quinone ring penetrates within the CD cavity, leaving the hydrocarbon chain outside. It is conceivable that the proximity of the IDE hydrocarbon chain to the sulfobutyl groups of the macrocycle limits (by steric hindrance) the electrostatic interaction of the negative sulfobutyl groups with the positive amino groups of CS. IDE was encapsulated with a good efficiency (E.E.) percentage; however, a low drug content (D.C.) percentage was observed due to the limited theoretical IDE/SBE-β-CD concentration that we could add during the NP preparation. As a matter of fact, a higher amount of the IDE/SBE-β-CD inclusion complex could not be used because large aggregates were obtained.

**Figure 2:**
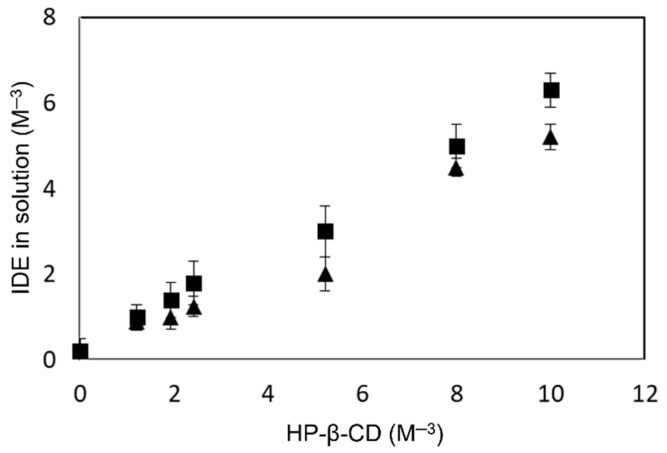
Phase solubility diagram of the IDE-HP-β-CD inclusion complex determined at pH 5.0 and 25.0 ± 0.1 °C. ∎ without CS and ▲ with CS.

To improve the encapsulation properties of the CS NPs, we overloaded the system by using the soluble IDE/HP-β-CD inclusion complex. The physical–chemical characterization of this inclusion complex was carried out in a previous paper [24]. HP-β-CD does not possess a charge; therefore, it does not interfere with the gelation process induced by SBE-β-CD. However, before preparing the overloaded CS NPs, the influence of CS on the complexing ability of HP-β-CD toward IDE was evaluated. Thus, solubility phase studies were performed at pH 5.0, in the absence and presence of a fixed amount of CS, corresponding to that used for NP preparation. The obtained profiles are showed in Figure 2. We observed, for both cases, a linear relation between the increase of the macrocycle concentration and the IDE solubility. Nevertheless, in the presence of CS, a small decrease of the stability constant value (K_C_) of the complex, from 6672 M^−1^ to 5154 M^−1^, was observed. This effect could be due to a steric hindrance produced by CS chains, which prevent the inclusion of IDE into CD cavity. However, the formation of an inclusion complex between HP-β-CD and CS chains cannot be excluded, based on a study carried out by other authors [52] that demonstrated the ability of native β-CD to include CS. In all cases, the negative influence exerted by CS on the complexing ability of HP-β-CD toward IDE was limited, and only a little reduction of the complexation efficiency (C.E.) was observed, passing from about 1.30 to 1.03 for the complex prepared in the absence or in the presence of CS, respectively.

The IDE/HP-β-CD inclusion complex was prepared in a 1:2 molar ratio by freeze-drying and added to a polycation or polyanion solution. As expected, no influence of HP-β-CD was exerted on the gelation process, obtaining small and homogeneous NPs at all of the used theoretical amounts. ζ was also not influenced, demonstrating once again the presence of positive CS chains on the NP surface (Table 2).

The encapsulation parameters characterizing overloaded CS NPs, reported in Table 3, turned out to be improved with respect to the non-overloaded ones. However, these parameters seem to be differently affected by the addition of the IDE/HP-β-CD inclusion complex to the polyanion (IDE/SBE-β-CD solution) or to the polycation solution (CS solution). On the one hand, a progressive reduction of the yield (%) and an increase in the E.E. (%) and D.C. (%) parameters were observed as the amount of the inclusion complex added in the polyanion phase increased. On the other hand, the addition of the increasing amount of the IDE/HP-β-CD inclusion complex to the polycation phase produced a progressive increase of the yield (%) at all IDE theoretical amounts; whereas, E.E. (%) and D.C. (%) increased until a theoretical IDE amount of 2.5 mg, after that, a reduction of the cargo ability of the NPs was observed.

**Figure 3:**
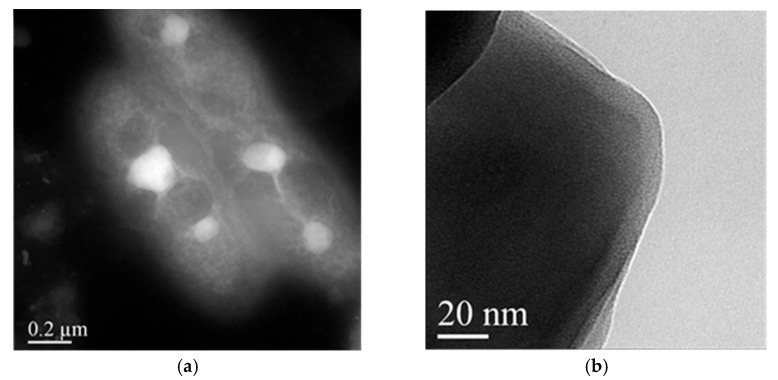
STEM images of non-overloaded CS NPs at different magnifications. Images are representative of at least three independent experiments. The bar width in the panels (**a**) 200 nm and (**b**) 20 nm indicated the size.

For the successive studies, we selected two formulations between the CS NPs pre-pared by adding IDE/HP-β-CD inclusion complex to the polyanion solution (CS NPs-PA) or to the polycation solution (CS NPs-PC). In both cases, we chose the formulation prepared starting from 2.5 mg of theoretical IDE. In the case of CS NPs-PC, the choice was done due to its higher yield (%), E.E. (%), and D.C. (%), with respect to the other formulations. Whilst for CS NPs-PA, our choice represents a compromise between the lower sizes and the higher yield (%), E.E. (%) and D.C. (%). Morphological characterizations of NPs were carried out by the scanning transmission electron microscopy (STEM) analysis. All formulations (non-overloaded CS NPs, overloaded CS NPs-PC, and overloaded CS NPs-PA) showed similar morphology. They were spherical, non-aggregated, and at high magnifications they showed dense cores made of CS chains electrostatically interacting with negative SBE-β-CD, surrounded by a less dense shell made by free CS chains [53], as confirmed by the positive ζ values of the NPs (see Table 1 and Table 2). This occurrence looks very interesting, since it can permit the adhesion of the systems to a negatively-charged nasal epithelium [54], representing the first step to the NP translocation to the brain. In Figure 3a,b, we show, as an example, the pictures of non-overloaded CS NPs at different magnifications. Similar images were obtained for overloaded CS NPs-PC and CS NPs-PA; they are shown in the Supplementary Materials (see Figures S1 and S2).

### 2.1. In Vitro Release Studies

**Figure 4:**
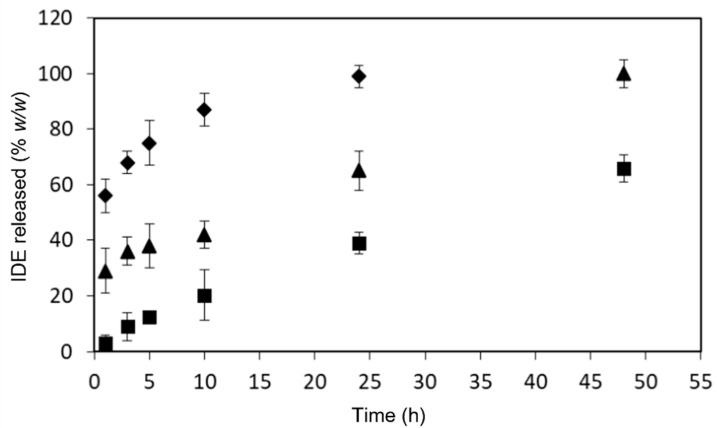
In vitro release profiles of IDE from NP formulations in PBS (pH 7.4), at 37.0 ± 0.5 °C. ∎ non-overloaded CS NPs; ▲ overloaded CS NPs-PC and ◆ overloaded CS NPs-PA.

In vitro release studies of IDE from overloaded CS NPs-PC and CS NPs-PA were carried out in comparison with the non-overloaded CS NPs in a phosphate buffer solution (PBS, pH 7.4). The obtained profiles are reported in Figure 4.

The non-overloaded CS NPs showed a sustained release of IDE that lasted beyond the experimental time. No burst effect was observed, and after 48 h from the beginning of the experiment, only 60% (*w*/*w*) of the drug was released. IDE was present in the NP matrix as the inclusion complex with SBE-β-CD. It is conceivable that, based on the morphology observed by the STEM analysis, IDE was homogeneously distributed within the core of NPs included into the macrocycle. As a consequence, the burst effect was avoided. Furthermore, we can hypothesize that IDE was released by the CS NPs as a free drug, and not as the inclusion complex, being the macrocycle involved in interactions with CS chains.

A different trend was observed for the overloaded formulations. Both systems showed a burst effect in the first hour of the experiment of about 60%, (*w*/*w*) and about 30% (*w*/*w*) for overloaded CS NPs-PA and overloaded CS NPs-PC, respectively. The burst effect observed for the overloaded formulations demonstrated the presence of the IDE/HP-β-CD inclusion complex on the NPs surface or in its proximity, particularly for CS NPs-PA. After that, the release of IDE from this latter formulation was fast and quantitative within 10 h. This occurrence can be explained taking into account the high solubility of the IDE/HP-β-CD inclusion complex, which was quickly released when overloaded CS NPs-PA were put in contact with the release medium. The IDE/HP-β-CD inclusion complex is likely more homogeneously distributed within the polymeric matrix of the overloaded CS NPs-PA; this produced a low burst effect in the first hour of the experiment with respect to the CS NPs-PA, and a sustained release of IDE for 48 h.

The release data were treated according to different kinetic models, as described in the Materials and Methods section. The obtained parameters (Table 4) revealed, for non-overloaded CS NPs and overloaded CS NPs-PC formulations, the best correlation (as indicated by R^2^ values) within the zero-order kinetic model, evidencing a constant release of IDE in time, as requested for an ideal delivery system. The CS NPs-PA showed the best correlation within the first order kinetic model, likely due to the high amount of the soluble IDE/HP-β-CD inclusion complex present on the NP surface, which produced the fast release at the beginning of the experiment. These results demonstrated the suitability of non-overloaded CS NPs and overloaded CS NPs-PC to the prefixed therapeutic objective, i.e., the sustained release of the drug, after targeting to the brain. Furthermore, all of the systems showed a good correlation with Hixson–Crowell model, evidencing that the release process involves erosion/diffusion and alterations in the sizes and surface areas of the NPs.

### 2.2. In Vitro Biological Studies

**Figure 5:**
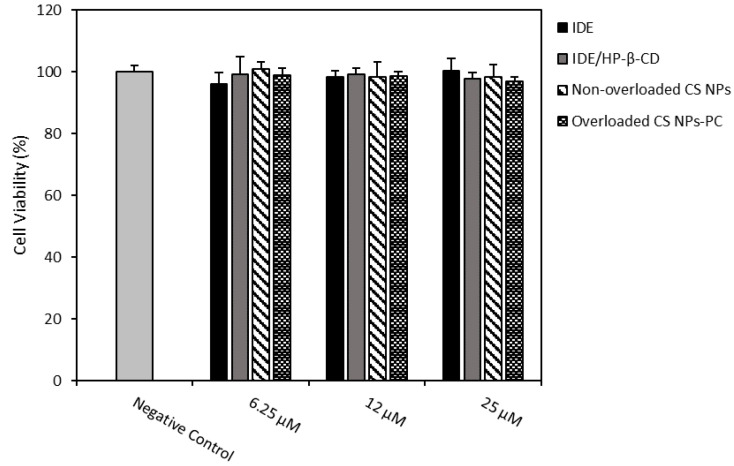
Effects on U373 cell viability of 24 h treatment with 6.25, 12, and 25 µM of IDE, IDE/HP-β-CD, non-overloaded CS NPs, and overloaded CS NPs-PC. Cell viability was measured using the MTT test. Results are presented as the means of three different experiments (six replicates for each point) ± standard deviation. No significant reduction in cell viability (%) was reported in the treated vs. untreated cells.

Based on the results obtained from the release studies, demonstrating a fast release for overloaded CS NPs-PA and a sustained release of IDE just for non-overloaded CS NPs and overloaded CS NPs-PC, we decided to carry out biological studies on these last formulations, comparatively to free IDE and the IDE/HP-β-CD inclusion complex. Even if the antioxidant activity of the IDE/HP-β-CD inclusion complex on U373 cells were already performed in our previous study [24], here, the need for comparation was because the free drug and inclusion complex were released by the overloaded CS NPs-PC. So, the effects of all species contacting with the cells were assayed. We evaluated the effects on U373 cell viability of 24 h treatment with 6.25, 12, and 25 µM of IDE, IDE/HP-β-CD inclusion complex, non-overloaded CS NPs, and overloaded CS NPs-PC, by methylthiazolyldiphenyl-tetrazolium bromide (MTT) test (Figure 5). None of the assayed samples negatively affected the U373 cell viability, which maintained equal to the control (cell viability higher than 95%). 

Antioxidant activity of free IDE, empty CS NPs, non-overloaded CS NPs, overloaded CS NPs-PC, and the IDE/HP-β-CD inclusion complex were assayed on U373 cells by inducing an oxidative stress with hydrogen peroxide (H_2_O_2_) and measuring the resulting LDH release by the cells. Before the oxidative stress, cells were pre-treated with the samples at a drug concentration of 6.25, 12, and 25 µM, to evaluate their protective effects against the H_2_O_2_-induced cell damage. 

**Figure 6:**
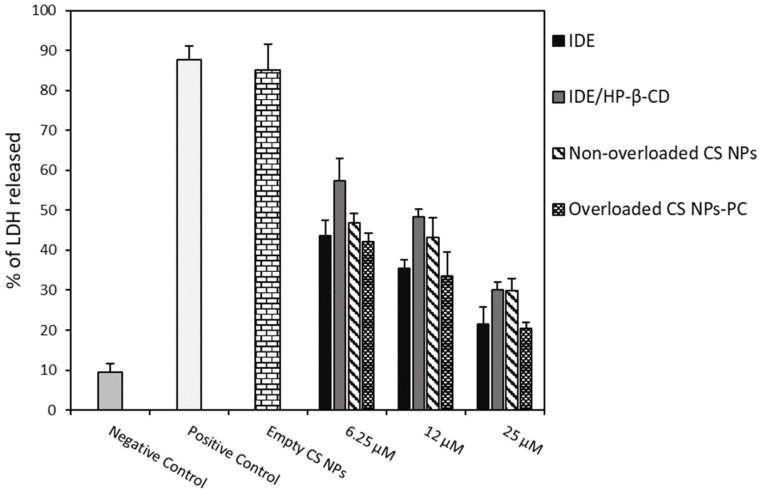
Protective effects of different concentrations of IDE, the IDE/HP-β-CD inclusion complex, non-overloaded CS NPs, and the overloaded CS NPs-PC on U373 cells against H_2_O_2_ damage. Cells were treated with all samples for 24 h. After that, they were incubated with H_2_O_2_ (700 μM) for 1 h. LDH release was assessed as described in Section 3. Results are presented as the means of three different experiments (six replicates for each point) ± standard deviation. No significant reduction in LDH release has been recorded from cells treated with all realized IDE-loaded formulations compared to the treated cells with free IDE.

As observed in Figure 6, empty CS NPs did not show any protective effects on U373 cells toward H_2_O_2_-induced damage, demonstrating a high LDH release, comparable to that observed for the non-pre-treated U373 cells (positive control). A similar result was previously obtained for free HP-β-CD; thus, it was not reported [24]. All of the assayed samples showed dose-dependent antioxidant effects. In particular, at the lowest dose, both CS NP formulations showed similar antioxidant activity to free IDE, and higher than the IDE/HP-β-CD inclusion complex. This trend slightly changed at the highest dose, observing similar antioxidant activity for the inclusion complex and non-overloaded CS NPs but always lower than the free drug and overloaded CS NPs-PC. In all cases, the overloaded CS NPs-PC showed similar antioxidant effects to that of free IDE. At first sight, this trend appears as a negative result; that is, no increase in the antioxidant activity of IDE-loaded CS NPs with respect to the free drug. However, some considerations permit other interpretations: (i) free IDE is soluble at the concentration used in this study and all of the assayed doses are available to interact with the cells; (ii) CS NPs slowly release IDE as a free drug (non-overloaded CS NPs) and/or as the IDE/HP-β-CD inclusion complex (overloaded CS NPs-PC), thus reducing the amount of free drug available to interact with the cells Actually, considering that after 24 h (the experimental time) only 38% (*w*/*w*) of free IDE and 60% (*w*/*w*) of free IDE or/and the IDE/HP-β-CD inclusion complex was released by non-overloaded and overloaded CS NPs-PC, respectively (see the release profiles in Figure 4), a lower antioxidant activity, with respect to free IDE, should be expected for both CS NP formulations. Thus, the comparable antioxidant activity observed for free IDE and CS NP formulations is a very positive result and can be explained by considering the permeabilizing action exerted by CS NPs on the biomembranes [55], which allows rapid entry of the released drug within the cells, so balancing the lesser amount of IDE contacting the cells. Moreover, the higher permeabilization of the cellular membrane may lead to a faster uptake of IDE-loaded NPs, allowing the release of IDE after cellular internalization and, thus, improving the antioxidant effect of the drug on the inner cellular membrane layer.

Regarding the lower antioxidant activity shown by the inclusion complex with respect to the free drug, we must consider that, as reported [24,56], the inclusion complex is not able to express the antioxidant activity of the drug; only the free IDE that is released from the complex, as a function of the Kc value, can show the antioxidant effects. We did not observe high differences because HP-β-CD is able to interact with the biomembranes, increase drug permeation [57], and balance the low amount of free IDE available to interact with cells.

**Figure 7:**
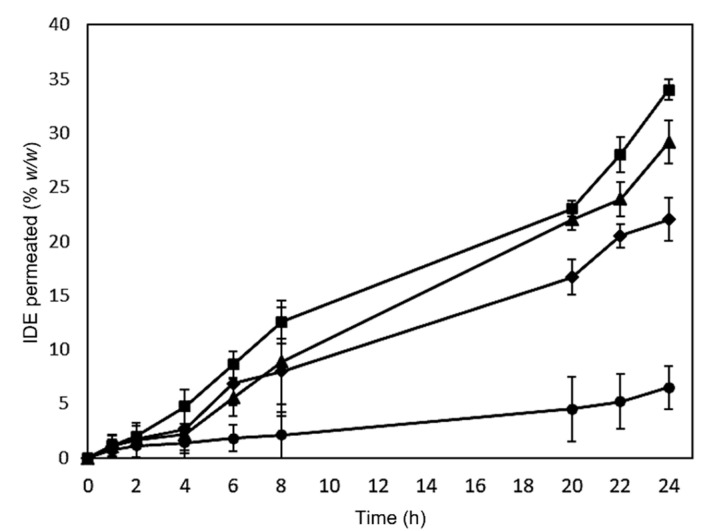
Permeation profiles across the excised bovine nasal mucosa of free IDE (circles), IDE/HP-β-CD inclusion complex (squares), non-overloaded CS NPs (diamonds), and the overloaded CS NPs-PC (triangles). The results are presented as the means of six different experiments ± standard deviation.

To confirm the ability of the formulations to improve the biomembrane permeation of IDE, we carried out experiments through excised bovine nasal mucosa mounted on Franz’s cells. The obtained results are showed in Figure 7.

All assayed formulations show higher IDE penetration through the mucosa with respect to free IDE in the following order: IDE < non-overloaded CS NPs < overloaded CS NPs-PC < IDE/HP-β-CD inclusion complex. Our previous studies [24], carried out for an experimental time of 4 h, already demonstrated the ability of HP-β-CD to improve the permeation through the nasal mucosa of IDE. Here, the experiment performed for a higher time confirmed this trend, which can be explained based on the increase of IDE water solubility because of the complexation into the macrocycle. We observed about 35% (*w*/*w*) and 5% (*w*/*w*) IDE permeation after 24 h treatment with the IDE/HP-β-CD inclusion complex and free IDE, respectively. In the biopharmaceutical classification, IDE is a class II drug, with inadequate solubility and good permeability when in a solution. Thus, its bioavailability is dissolution rate-dependent. IDE, similar to most drugs, is absorbed by passive diffusion through the biomembranes, by partition from the aqueous environment to the outermost membrane layer [58]. It is important that the aqueous external layer of the membrane is saturated by the drug (i.e., the highest thermodynamic potential) [59] since a high amount of the dissolved drug at the membrane external layer will give rise to high permeation through the membrane. In this experiment, free IDE was assayed as a suspension, and due to its limited water solubility, its absorption was very low. Whilst high-water soluble IDE/HP-β-CD inclusion complex produces a complex-saturated layer in contact with the biomembrane [60], and the drug released by the complex, as a function of the K_C_ value, was promptly absorbed due to its high lipophilicity. Moreover, HP-β-CD is reported to be responsible for a cholesterol depletion by the viable membranes, producing a destabilization of the bilayer and increasing the permeability to the drug [61]. 

Concerning the two NP formulations, we observed a comparable amount of IDE permeated through the mucosae until 8 h. After this period, a difference in the IDE permeation was evident for the two formulations, which maintained in all cases lower than the one revealed in the case of inclusion complex. Actually, based on the obtained release profiles (see Figure 4), a more marked difference between the two formulations was expected. Thus, it is conceivable that both overloaded and non-overloaded NP similarly interact with the mucosae, due to their similar surface charge, about + 28 mV (Table 1 and Table 2). The positive charge, due to the presence of free CS chains on the surface of the NPs, makes them able to efficiently attach to cells [62], altering the membrane permeability and improving the drug permeation [63]. Furthermore, the cellular uptake of NPs cannot be excluded or improved by the interaction between NPs and biomembranes [64,65]. In this way, a similar amount of overloaded and non-overloaded NPs could penetrate the mucosa in the first hours of the experiment. After this time, the permeation probably depends on the release rate of IDE from the NPs, lower for non-overloaded NPs, corresponding to a lower IDE permeation. 

Regarding the higher IDE permeation observed for the inclusion complex, with respect to the CS NP formulations, this was expected due to the slow release of the drug from these systems.

In order to verify this hypothesis, the mucosae used for permeation studies was homogenized and IDE was extracted and determined. In Table 5, we show the amount of the drug accumulated after 24 h of treatment with the different formulations. We observed a very low amount of free IDE accumulated in the mucosa. These results are in agreement with the observed poor availability of the free drug to permeation. As expected, high accumulation of IDE was observed after treatment with the soluble inclusion complex, demonstrating the strong influence exerted by IDE solubilization by HP-β-CD on the bioavailability of the drug. Regarding the NP formulations, we observed, after 24 h of treatment, a similar cumulative amount of IDE into the mucosae, i.e., about 26% and 28% for non-overloaded CS NPs and the overloaded CS NPs-CA, respectively. These values do not reflect the difference of IDE permeated from the two formulations after 24 h treatment, i.e., 21% and 29% for non-overloaded CS NPs and the overloaded CS NPs-CA, respectively. This trend could confirm that penetration of NPs into the mucosa is only related to the characteristic properties (sizes and surface charges) of the NPs themselves, and not to those of the encapsulated payload (free drug or IDE/HP-β-CD inclusion complex). Obviously, the properties of the payload and its distribution into the polymeric matrix influence its release from the NPs, and then its transfer from mucosa to the receptor compartment, giving rise to a faster transfer for soluble IDE/HP-β-CD inclusion, with respect to free IDE.

**Figure 8:**
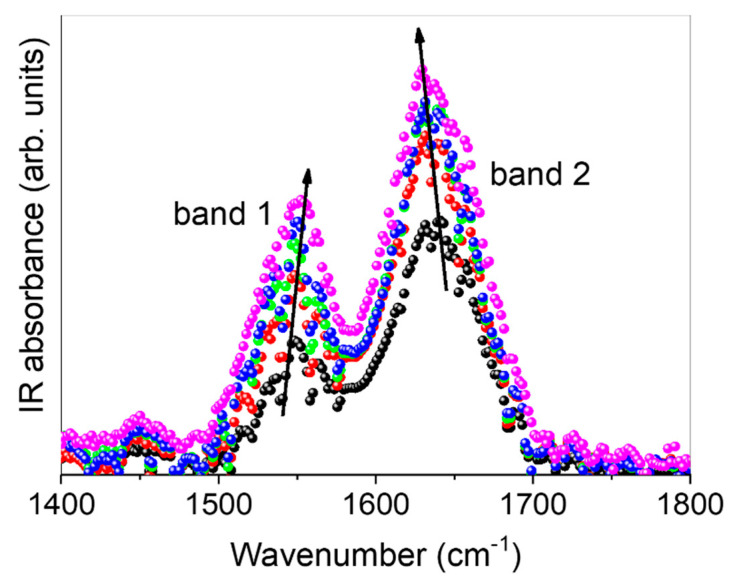
Experimental FT-IR spectra for untreated excised bovine nasal mucosa (black circles), and for the mucosae treated for 24 h with free IDE (red circles), IDE/HP-β-CD inclusion complex (pink circles), the non-overloaded (green circles), and overloaded CS NPs (blue circles). Arrows are guides for the eyes. See text for details.

Finally, the ability of the macrocycle and CS NPs to interact with the excised bovine nasal mucosa was investigated through FT-IR studies. It is reasonable to assume that the intermolecular interactions were mainly due to hydrogen bonds. In light of this, we focused our attention on the 1400–1800 cm^−1^ wavenumber spectral range, to which IR-active vibrational modes (sensitive to breaking/reformation of hydrogen bonds) belong (Figure 8).

In this region, the FT-IR profile of all investigated samples exhibits a very intense broad band (band 1) centered at about 1548 cm^−1^, assigned to Amide II, mainly resulting from δ(N-H) bending modes, convoluted to the bending modes of the C-H groups. Moreover, the intense, broad band (band 2) found at about 1640 cm^−1^ is mainly associated with the Amide I band mainly due to ν(C=O) stretching vibration amide groups, probably convoluted to the bending vibrational mode of water molecules, generally indicated as δ(HOH). As can be seen, the intensity of band 2 increases, passing from untreated mucosa to that treated with, in order, free IDE, non-overloaded CS NPs, overloaded CS NPs, and, finally, the IDE/HP-β-CD inclusion complex. At the same time, its center-frequency is slowly down-shifted. These spectral modifications can be explained in terms of an always more co-operative HB scheme in which water molecules surrounding the C=O groups are involved. This implies that the electrostatic environment experienced by these chemical groups is such that it reduces their overall dipole moment [66]. On the other side, band 1 is observed to increase in intensity, with an up-shift in the wavenumber, passing from untreated mucosa to that treated with, in order, free IDE, non-overloaded CS NPs, overloaded CS NPs, and, finally, the IDE/HP-β-CD inclusion complex. This occurrence suggests that these vibrations become progressively less hindered. This could be due to an induced breaking of non-conventional hydrogen bond interactions with water molecules, of the type N-H· · · O-H (or C-H· · · O-H), in which NH (or CH) groups may be involved [67]. 

## 3. Materials and Methods

### 3.1. Materials

Idebenone [IDE; 2,3-dimethoxy-5-methyl-6(10-hydroxydecyl)1,4-benzoquinone, C_19_H_30_O_5_, MW 338.44 Da], low molecular weight chitosan (CS; (C_12_H_24_N_2_O_9_)_n_; 75–85% deacetylated; MW, 50,000–190,000 Da based on viscosity; viscosity: 20–300 cps, 1% solution in 1% acetic acid), 2-hydroxypropyl-β-cyclodextrin (HP-β-CD; 0.6 molar substitution, average MW 1380 Da), D-(+)-trehalose dihydrate were supplied by Sigma–Aldrich (Milan, Italy). Sulfobutyl ether-β-cyclodextrin (SBE-β-CD; CAPTISOL^®^, CyDex Pharmaceuticals, Inc., Shawnee, KS, USA; mean sulfobutyl replacement 7; average MW 2162 Da) were kindly provided by CyDex Pharmaceutical (Lenexa, Kansas City, MO, USA). The water used in the study was twice distilled, de-ionized, and finally filtered (0.22 μm Millipore^®^ GSWP filters, Bedford, FC, USA).

### 3.2. Preparation of the IDE/SBE-β-CD and the IDE/HP-β-CD Inclusion Complexes

The IDE/SBE-β-CD and the IDE/HP-β-CD inclusion complexes were prepared by a freeze-drying method in 1:2 molar ratio. CDs (0.00345 × 10^−3^ M) were solubilized, at room temperature, in 8 mL of water and added, under stirring, drop by drop of an IDE-methanol solution (0.00172 × 10^−3^ M, 2 mL). The solutions were poured into freeze-drying flasks and freeze-dried for 72 h (VirTis Gardiner, USA BenchTop K Series Freeze Dryers, Gardiner, NY, USA).

### 3.3. Phase Solubility Studies of IDE

Increasing amounts of HP-β-CD (0–10 × 10^−3^ M) were solubilized in aqueous solutions at pH 5, into 10 mL vials, in the absence or presence of a fixed amount of CS (10 mg), then solid IDE was added in amount exceeding its intrinsic solubility. Vials were poured into a thermostatic bath at 25.0 ± 0.1 °C (Telesystem stirring bath thermostat 15.40 with Telemodul 40 C control unit) under stirring for 48 h. After that, the suspensions were filtered through Sartorius Minisart^®^-SRP 15 PTFE 0.20 μm filters to eliminate the excess IDE. The filtrate, containing the IDE/HP-β-CD complex and CS in the solution, was added to acetonitrile to precipitate CS and the CD, and filtered again; then, IDE was quantified into the solutions by HPLC (Shidmazu LC-10 AD VP, Delhi, India). The 1:1 stability constants (K_C_) of the IDE/HP-β-CD inclusion complex in the presence or absence of CS were determined by the Higuchi and Connors method [68], reporting in a graph the amount of IDE in the solution as a function of the CD concentration or CD + CS concentration. The following equation was used:

(1) $$K_{C}=\alpha /S_{0}(1-\alpha ),$$

where α is the slope of the obtained linear plot and S0 is the solubility of IDE in the aqueous solution at pH 5 [68].

The complexation efficiency (C.E.) was determined according to the following equation [69]:

(2) $$C.E.=S_{0}\cdot K_{C}$$

### 3.4. Preparation of CS NPs

CS NPs were prepared by ionotropic gelation of CS, using as gelling agents free SBE-β-CD (empty CS NPs) or the IDE/SBE-β-CD inclusion complex (non-overloaded CS NPS). Briefly, CS (13 mg) was solubilized under magnetic stirring in 14 mL of an aqueous acetic acid solution (1%, *v*/*v*), previously adjusted to pH 5, with the addition of 2 M NaOH drops. After that, 2 mL of aqueous solution (pH 5), containing free SBE-β-CD (13 mg) or the same amount in the IDE/SBE-β-CD inclusion complex (1:2 molar ratio), was added dropwise, under magnetic stirring, to the CS solution. The obtained colloidal suspensions were magnetically stirred for 30 min to allow the complete formation of the systems. After that, the NPs were isolated by centrifugation at 18,000 rpm for 30 min with a Beckman Optima™ XL-100K ultracentrifuge (Beckman Coulter Italy, Milan, Italy). The pellets, constituted by empty CS NPs or non-overloaded CS NPs, were resuspended in 1 mL of water containing 5% trehalose as a lyoprotectant agent, and freeze-dried for 72 h (VirtTis Benchtop K Instrument, SP Scientific, Gardiner, MT, USA). The supernatant derived from centrifugation of empty CS NPs was discarded, whilst the supernatant derived from centrifugation of non-overloaded CS NPs were collected and used for indirect dosage of the drug within the NPs (see Section 3.6 for details). 

To prepare the overloaded CS NPs, different amounts of the solid IDE/HP-β-CD inclusion complex, containing 0.5, 1, 1.5, and 2 mg of IDE, were solubilized into the polycation (CS in 14 mL of pH 5) aqueous solution or the polyanion (IDE/SBE-β-CD inclusion complex, 1:2 molar ratio, in 2 mL of pH 5) aqueous solution, prepared as previously described. Thus, NPs were obtained using the procedure already described to prepare empty CS NPs and non-overloaded CS NPs. The pellets of overloaded CS NPs, obtained after centrifugation, were resuspended in 1 mL of water, containing 5% trehalose, and freeze-dried. The supernatants were used for indirect dosages of IDE within the NPs (see Section 3.6 for details). 

### 3.5. Characterization of CS NPs

Freeze-dried NPs were weighted to determine the yield (%), following the formula:

Yield (%) = (Effective yield/Theoretical yield) × 100.(3)

The encapsulation efficiency (E.E.) and the drug content (D.C.) percentage of IDE-loaded NPs were indirectly determined by quantification of IDE in the supernatant. Briefly, the supernatant was added at an equal volume to the acetonitrile. In this way, we precipitated the excess of CS and CDs because of their low solubility in this organic solvent. The suspension was filtered (LLG-Syringe filters Spheros, Meckenheim, Germany, 0.45 µm Ø 25 mm), and IDE present in the solution was determined by the HPLC analysis. The following equations were used:

E.E. (%) = [(Theoretical IDE in mg − IDE in supernatant in mg)/Theoretical IDE in mg] × 100 (4)

D.C. (%) = [(Theoretical IDE in mg − IDE in supernatant in mg)/Weight of recovered NPs] × 100(5)

The lyophilized CS NPs were redispersed and the mean hydrodynamic radius (R_H_) was determined by using a Zetasizer Nano ZS (Malvern Instrument, Malvern, UK), utilizing a noninvasive back-scattering (NIBS) technique. The measurements were performed at room temperature (25 ± 1 °C), at an angle of 173° with respect to the incident beam. A non-negative least-squares algorithm was used to achieve the deconvolution of the measured correlation curve. The zeta potential values (ζ) were measured by using a Zetasizer Nano ZS (Malvern Instrument, Malvern, UK) with a 633 red laser and a power of 5.0 mW. Three separate measurements of three different batches were carried out; the ζ values are expressed as mean ± standard deviation (S.D.). A volume (100 μL) of each sample was diluted with water (20 mL) and analyzed. The Smoluchowsky equation (nominal value of the Smoluchowsky constant, 1.5) was used to determine the ζ values, on the basis of electrophoretic mobility. The ζ values are reported as the average of 10 experiments.

Morphological characterization was carried out using a Jeol JEM 2010F (JEOL, Ltd., Tokyo, Japan) transmission electron microscope (TEM) at 200 kV of accelerating voltage.

### 3.6. In Vitro Release of IDE from NPs

The release profiles of IDE from overloaded and non-overloaded CS NPs were determined using dynamic Franz diffusion cells (LGA, Berkeley, CA, USA). The diffusional surface area was 0.75 cm^2^, and the volume of the receptor compartment was 4.75 mL. Between the donor and receptor compartment, a synthetic cellulose membrane was placed (molecular cut-off 8000 Da). Free IDE, or IDE in non-overloaded CS NPs and overloaded CS NPs (0.5 mg), were suspended in 500 µL of PBS (pH 7.4), then an aliquot of 200 µL of each sample was placed in the donor compartment. The receptor compartment was maintained at 37.0 ± 0.1 °C, under magnetic stirring and, to ensure the sink condition, it was filled with PBS (pH 7.4)/ethanol solution (60/40, *v*/*v*). At different times (1, 3, 5, 10, 24, and 48 h), an aliquot (500 µL) of the receptor phase was withdrawn and analyzed by HPLC for IDE quantification. The withdrawn volume was replaced by a fresh mixture of PBS (pH 7.4)/ethanol solution (60/40, *v*/*v*). Three experiments were carried out and the results are presented as mean ± standard deviation (S.D.).

The data obtained from the in vitro release studies were analyzed by using different equation models, i.e., zero-order equation, plotting cumulative percentage of the released drug vs. time, first-order equation, plotting log cumulative percentage of the remaining drug vs. time, Higuchi equation, plotting cumulative percentage of the released drug vs. the square root of time, and the Hixson–Crowell cube root law, plotting the cubed root of the initial percent concentration minus the cubed root of the percentage remaining vs. time [70,71,72]. Among them, the one exhibiting the highest correlation coefficient (R^2^) was considered the “best model”.

### 3.7. HPLC Analysis 

HPLC analyses were carried out by using a Shimadzu apparatus consisting of an HPLC LC-20 AB Prominence and a SPD-20A Prominence UV–vis detector (Shimadzu Italia, Milano, Italy). The analyses were performed at room temperature (25 °C), using a Discovery C-18 column (5 μm, 250 mm × 4.6 mm I.D.) (Supelco^®^). IDE was detected at 280 nm, and isocratically eluted with acetonitrile/water (75/25, *v*/*v*) at a flow rate of 1 mL/min. The linear regression coefficient, in the range between 0.5 µg/mL to 50 µg/mL, was 0.99998 (*n* = 6).

### 3.8. FT-IR Analysis of Excised Nasal Mucosae

Thin slides of the excised bovine nasal mucosa treated for 24 h with free IDE, the IDE/HP-β-CD inclusion complex, and the non-overloaded and overloaded CS NPs, about 30 µm thick, were obtained by using a standard cryostat methodology with a freezer microtome, without any embedding procedure [73,74]. This method ensured the prevention of any unwanted contribution in the investigated range that could be due to substances eventually being used for fixing. The slides were placed on KBr pellets, transparent in the analyzed wavenumber range, from 400 to 4000 cm^−1^. FT-IR measurements were performed at room temperature on a Bomem DA8 Fourier transform spectrometer, operating with a Globar source, in combination with a KBr beam splitter and a DTGS/KBr detector. The spectra were recorded with a resolution of 4 cm^−1^ in the dry atmosphere. Suitable reproducibility and the signal-to-noise ratio were obtained thanks to the 100 repetitive scans automatically added. Spectra were normalized (baseline adjustment) by using Spectracalc software GRAMS (Galactic Industries, Salem, NH, USA). Any further mathematical correction was used.

### 3.9. In Vitro Biological Studies

#### 3.9.1. Cell Cultures

Human U373 glioblastoma culture cells were incubated (Water-Jacketed CO_2_ Incubator, Thermo Scientific, Karlsruhe, Germany) in plastic culture dishes (100 mm × 20 mm) at 37.0 ± 0.1 °C (5% CO_2_) using a Dulbecco’s Modified Eagle Medium (DMEM) with glutamine, enriched with penicillin (100 UI/mL), streptomycin (100 µg/mL), amphotericin B (250 µg/mL), and fetal bovine serum (FBS) (10% *v*/*v*), to promote their adhesion to the plate. Fresh medium was substituted every 48 h until 80% confluence was reached, and then trypsin (2 mL) was added for cell detaching. After that, the cells were collected into a centrifuge tube containing 4 mL of the culture medium. To collect all remaining cells, the dishes were further washed with 2 mL of PBS (pH 7.4), and cells were centrifuged at 1500 rpm for 5 min by Megafuse 1.0 (Heraeus Sepatech, Osterode/Harz, Germany). Finally, DMEM was used as the medium to resuspend cells, which were seeded in culture dishes to perform in vitro studies

#### 3.9.2. In Vitro Cytotoxicity Assays

To evaluate cytotoxicity of free IDE, the IDE/HP-β-CD inclusion complex, and overloaded and non-overloaded CS NPs, a methylthiazolyldiphenyl-tetrazolium bromide (MTT) test was carried out. The U373 cells were plated in 96-multi-well dishes (8 × 103 cells/0.1 mL) and treated with different samples containing 6.25, 12, and 25 µM of IDE. The cells were incubated for 24, 48, or 72 h, and then 10 µL of tetrazolium salts (5 mg/mL in PBS, pH 7.4) were added to the dishes and incubated for 3 h. At the end of the incubation, cell viability was calculated by an ELISA microplate reader (BIO-RAD, xMark^TM^Microplate Absorbance Spectrophotometer, Hercules, CA, USA) at λ_abs_ = 570 nm and λ_abs_ = 670 nm according to the following equation:

cell viability (%) = AbsT/AbsC × 100 (6)

where AbsT/AbsC represents the ratio of cells treated (AbsT) and untreated control cells (AbsC). Three independent experiments were performed and data are reported as the mean of results ± standard deviation (S.D.).

#### 3.9.3. Evaluation of Antioxidant Activity

Antioxidant activity was assayed by measuring lactic dehydrogenase (LDH) release. The U373 cells were placed in 96-well culture dishes (8 × 103 cells/0.1 mL) and treated with different samples (IDE/HP-β-CD inclusion complex, overloaded and non-overloaded CS NPs), containing 6.25, 12, and 25 µM of IDE for 24 h, and then incubated with hydrogen peroxide (700 µM) for 1 h. The effects on cell cultures were then evaluated by LDH release using a suitable Pierce LDH cytotoxicity assay kit. The LDH activity was detected using a spectrophotometer, setting λ = 680 nm and λ = 450 nm, measuring the ability of LDH to convert nicotinamide adenine dinucleotide (NAD) to reduced nicotinamide adenine dinucleotide (NADH). The percentage of LDH release was obtained from the following equation:

(7) $$\mathrm{\%LDH release}=\frac{\mathrm{Compound treated LDH activity}-\mathrm{Spontaneous LDH activity}}{\mathrm{Maximum LDH activity}-\mathrm{Spontaneous LDH activity}}\times 100,$$

where Compound treated LDH activity and Spontaneous LDH activity stands for LDH released by the treated cells and LDH released from the control cells, respectively. Maximum LDH activity stands for the maximum amount of LDH released from cells that underwent lysis. The percentage of LDH released was finally calculated subtracting the 690 nm absorbance value (considered as the background) from the 490 nm absorbance value. Results are presented as the mean values of three experiments ± standard deviation.

#### 3.9.4. Permeation Studies through Excised Nasal Mucosa

Bovine nasal mucosa was obtained from just slaughtered healthy animals (mean age 15 ± 3 months). Sections were bled using a mixture of PBS (pH 7.4) and heparin. Permeation studies were performed using Franz diffusion cells (LGA, Berkeley, CA, USA) that are characterized by a diffusion area of 0.75 cm^2^ and a nominal receiving volume of 4.75 mL. Mucosa samples were interposed between the donor (upper part) and receptor (lower part) compartment of Franz cells. The receptor was filled with a mixture of PBS (pH 7.4)/ethanol (60:40 *v*/*v*) to maintain sink conditions, while 200 µL of free IDE or different formulations containing IDE (50 µM) were added into the donor compartment. Franz cells were maintained at 37 ± 0.5 °C and samples were withdrawn from the receptor at scheduled times (1, 2, 4, 6, 8, 20, 22, 24 h), replacing the volume with a fresh solution of the same receptor phase that has been heated at 37 ± 0.5 °C. The integrity of nasal mucosa samples was investigated before and after permeating studies through the measurement of transepithelial electrical resistance (TEER), which was 41 ± 15 Ω cm^2^. Mucosa samples obtained from permeation studies were gently washed to remove any remaining formulation and homogenized for 5 min in a mixture of water/methanol (1:1 *v*/*v*, final volume 10 mL) using Ultra-Turrax 1 IKA (IKA1 Werke GmbH & Co. KG, Staufen, Germany) to determine the amount of IDE accumulated in the tissue. The supernatant was then collected, and the solvent was removed under reduced pressure. The obtained residues were resuspended in 2 mL of methanol to solubilize IDE. Finally, the suspension was filtered (using 0.2 µm Millipore Filter) and injected to HPLC for analytical detection of IDE, recovering 97% IDE (*w*/*w*). Six replicates were performed and the results are reported as the mean value ± SD.

#### 3.9.5. Statistical Analysis

All data are shown as the mean of the values obtained from the study ± standard deviation. The statistical analysis comparing different groups was performed by a one-way ANOVA analysis with a Bonferroni post hoc test. A *p* value < 0.05 was considered statistically significant [75].

## 4. Conclusions

In this work, we efficiently developed CS NPs for the potential nose-to-brain targeting of IDE. CS NPs were prepared by ionotropic gelation of CS, using SBE-β-CD (empty CS NPs) or the IDE/SBE-β-CD inclusion complex (non-overloaded CS NPs) as gelling agents. We obtained CS NPs with about 140 nm of hydrodynamic radius, and a positive ζ value (about +28 mV), able to guarantee the interaction of CS NPs with the negatively-charged nasal epithelium. CS NP formulation showed good encapsulation efficiency (about 40%) but low drug content (about 3%). To improve the encapsulation parameters, we overloaded the system by using different amounts of the highly soluble IDE/HP-β-CD inclusion complex. The complex was added to the polycation solution (CS NPs-PC) or to the polyanion solution (CS NPs-PA) and the properties of these formulations were evaluated in comparison to the non-overloaded CS NPs. All formulations showed positive ζ values as well as higher encapsulation efficiency and drug content, with respect to the non-overloaded CS NPs. The release profiles obtained for the realized formulations showed a sustained release of IDE from non-overloaded CS NPs (longer than 48 h, without a burst effect) and from CS NPs-PC (with a burst effect of about 30% and complete release within 48 h), showing their potentiality for nose-to-brain targeting of IDE. In vitro studies on U373 cells demonstrated the ability of CS NP formulations to enhance the penetration of IDE within the cells, so balancing the lesser amount of IDE contacting the cells, with respect to the free drug, due to its slow release from the formulations. Ex vivo studies on excised bovine nasal mucosa and FT-IR investigations of the inter-molecular interactions that occur between the carrier and the mucosa, showed higher permeation/interaction of IDE-loaded CS NPs with respect to free IDE.

Although more in vitro and ex vivo/in vivo studies are required, our results suggest that cyclodextrin based-CS NPs could be a potential system for the nose-to-brain delivery of IDE for the treatment of neurological disorders.

## Figures and Tables

**Table 1:** Hydrodynamic radius (R_H_), polydispersity index (P.I.), zeta potential (ζ), Yield (%), encapsulation efficiency (E.E.) (%), and drug content (D.C.) (%) of CS NPs produced by ionotropic gelation of CS with SBE-β-CD (empty CS NPs) and the IDE/SBE-β-CD inclusion complex (1:2 molar ratio) (non-overloaded CS NPs). The values are expressed as the mean of six batches ± standard deviation (S.D.).

Sample	Theoretical IDE Amount (mg) *	R_H_ (nm) ± S.D.	P.I. ± S.D.	ζ (mV) ± S.D.	Yield (%) ± S.D.	E.E. (%) ± S.D.	D.C. (%) ± S.D.
Empty CS NPs	-	138.28 ± 2.36	0.181 ± 0.056	+27.3 ± 2.5	75.12 ± 1.82	-	-
Non-overloaded CS NPs	1	140.08 ± 3.71	0.169 ± 0.068	+28.2 ± 1.9	53.2 ± 2.91	42.10 ± 2.03	2.93 ± 0.62

* It is the amount of IDE within the IDE/SBE-β-CD inclusion complex.

**Table 2:** Hydrodynamic radius (R_H_), polydispersity index (P.I.), and zeta potential (ζ) of CS NPs prepared using the IDE/SBE-β-CD inclusion complex (1:2 molar ratio) as a gelling agent and overloaded with different concentrations of the IDE/HP-β-CD inclusion complex (1:2 molar ratio) (overloaded CS NPs). The values are expressed as the mean of six batches ± standard deviation (S.D.).

Sample	Theoretical IDE Amount (mg) *	IDE/HP-β-CD Inclusion Complex Added to Polyanion Solution			IDE/HP-β-CD Inclusion Complex Added to Polycation Solution		
		R_H_ (nm) ± S.D.	P.I. ± S.D.	ζ (mV) ± S.D.	R_H_ (nm) ± S.D.	P.I. ± S.D.	ζ (mV) ± S.D.
Overloaded CS NPs	1.5	145.12 ± 1.36	0.206 ± 0.029	+29.0 ± 1.9	141.56 ± 3.12	0.146 ± 0.051	+30.2 ± 1.5
	2	164.08 ± 0.80	0.231 ± 0.079	+27.1 ± 0.9	139.08 ± 4.21	0.205 ± 0.029	+28.8 ± 2.3
	2.5	171.10 ± 4.69	0.169 ± 0.091	+28.1 ± 2.0	136.38 ± 0.91	0.224 ± 0.014	+29.3 ± 0.9
	3	189.24 ± 2.71	0.112 ± 0.043	23.0 ± 1.7	146.94 ± 5.10	0.201 ± 0.058	+30.1 ± 1.8

* It is the sum of the drug complexed with SBE-β-CD and HP-β-CD.

**Table 3:** Yield percentage, encapsulation efficiency (E.E.), and drug content (D.C.) of CS NPs prepared using the IDE/SBE-β-CD inclusion complex (1:2 molar ratio) as a gelling agent and overloaded with different concentrations of the IDE/HP-β-CD inclusion complex (1:2 molar ratio) (overloaded CS NPs). The values are expressed as the mean of six batches ± standard deviation (S.D.).

Sample	Theoretical IDE Amount (mg) *	IDE/HP-β-CD Inclusion Complex Added to Polyanion Solution			IDE/HP-β-CD Inclusion Complex Added to Polycation Solution		
		Yield (%) ± S.D.	E.E. (%) ± S.D.	D.C. (%) ± S.D.	Yield (%) ± S.D.	E.E. (%) ± S.D.	D.C. (%) ± S.D.
Overloaded CS NPs	1.5	47.02 ± 4.26	43.90 ± 4.32	4.44 ± 0.98	54.36 ± 2.41	48.25 ± 1.72	4.21 ± 1.01
	2	45.78 ± 3.69	45.91 ± 3.11	5.51 ± 1.21	56.63 ± 1.11	62.35 ± 6.13	6.09 ± 0.87
	2.5	43.12 ± 5.01	51.81 ± 2.13	7.44 ± 1.26	57.23 ± 7.01	72.74 ± 1.91	7.85 ± 1.20
	3	40.25 ± 6.32	55.25 ± 3.17	9.17 ± 2.01	64.01 ± 2.14	65.22 ± 5.12	6.80 ± 0.81

* It is the sum of the drug complexed with SBE-β-CD and HP-β-CD.

**Table 4:** Regression coefficient (R^2^) and rate constant (K_i_, i = 0, 1, H, HC) of IDE release from non-overloaded CS NPs and overloaded CS NPs-PC and CS NPs-PA, according to different kinetic models.

Samples	Zero Order Model		First Order Model		Higuchi Model		Hixson–Crowell Model	
	R^2^	K_0_ (h^−1^)	R^2^	K_1_ (h^−1^)	R^2^	K_H_ (h^−1/2^)	R^2^	K_HC_ (h^−1/3^)
Non-overloaded CS NPs	0.9907	1.2837	0.9856	0.0219	0.9851	10.254	0.9924	0.1067
Overloaded CS NPs-PC	0.9789	1.5525	0.8770	0.0855	0.9568	12.293	0.9052	0.0804
Overloaded CS NPs-PA	0.8579	1.6706	0.9946	0.1802	0.9630	10.871	0.9921	0.0272

**Table 5:** Cumulative IDE amount (%, *w*/*w*) detected into the excised bovine nasal mucosa after 24 h treatment with free IDE or IDE in different formulations. Results are presented as the means of six different experiments ± standard deviation.

Sample	Cumulative IDE (% *w*/*w*)
Free IDE	3.78 ± 2.11
IDE/HP-β-CD inclusion complex	34.56 ± 2.82
Non-overloaded CS NPs	25.98 ± 1.88
Overloaded CS NPs-PC	27.67 ± 3.15

## Data Availability

Data is contained within the article and Supplementary Materials.

## Supplementary Materials

The following supporting information can be downloaded at: https://www.mdpi.com/article/10.3390/ph15101206/s1, Figure S1: STEM images of overloaded CS NPs-PA at different magnification. Figure S2: STEM images of overloaded CS NPs-PC at different magnification.
