# Supplementary material for: Chitosan/Cyclodextrin Nanospheres for Potential Nose-to-Brain Targeting of Idebenone
Source: Pharmaceuticals (Basel). 2022 Sep 28;15(10):1206. doi: 10.3390/ph15101206 (PMC9612377; doi:10.3390/ph15101206)
Supplement: Supplementary file 1 [file pharmaceuticals-15-01206-s001.zip › pharmaceuticals-1849069-supplementary.pdf]

pharmaceuticals

MDPI

SUPPLEMENTARY MATERIAL

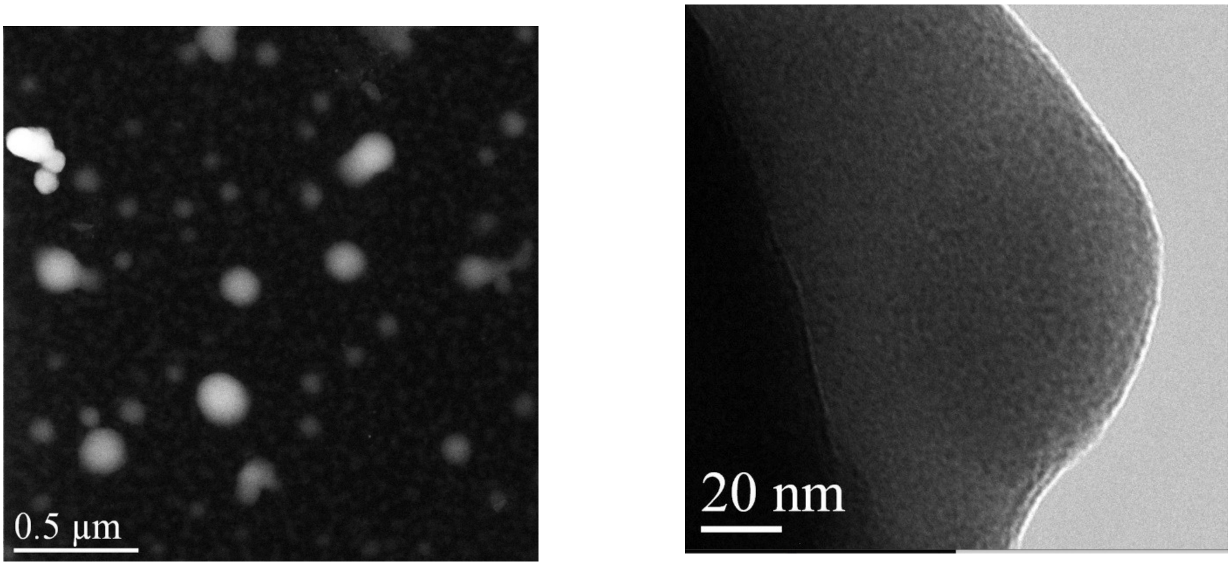

(**a**) (**b**)

Figure S1. STEM images of overloaded CS NPs-PA at different magnification.

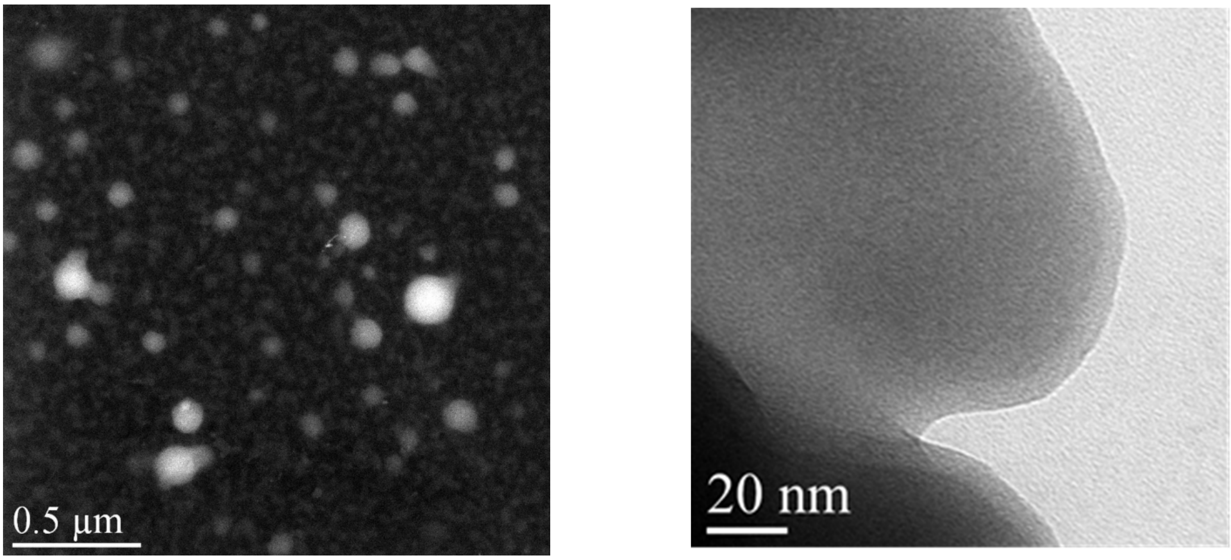

(**a**) (**b**)

Figure S2. STEM images of overloaded CS NPs-PC at different magnification.
